# Regulation of Bacterial DNA Packaging in Early Stationary Phase by Competitive DNA Binding of Dps and IHF

**DOI:** 10.1038/srep18146

**Published:** 2015-12-14

**Authors:** Sin Yi Lee, Ci Ji Lim, Peter Dröge, Jie Yan

**Affiliations:** 1 Mechanobiology Institute, National University of Singapore, Singapore 117411, Singapore; 2 Department of Physics, National University of Singapore, Singapore 117551, Singapore; 3 Center for Bioimaging Sciences, National University of Singapore, Singapore 117557, Singapore; 4 Division of Molecular Genetic and Cell Biology, School of Biological Sciences, Nanyang Technological University, Singapore 637551, Singapore; 5 NUS Graduate School for Integrative Sciences and Engineering, Singapore 117456, Singapore

## Abstract

The bacterial nucleoid, a bacterial genome packed by nucleoid binding proteins, forms the physical basis for cellular processes such as gene transcription and DNA replication. Bacteria need to dynamically modulate their nucleoid structures at different growth phases and in response to environmental changes. At the nutrients deficient stationary phase, DNA-binding proteins from starved cells (Dps) and Integration host factors (IHF) are the two most abundant nucleoid associated proteins in *E. coli*. Yet, it remains unclear how the nucleoid architecture is controlled by the interplay between these two proteins, as well as the nucleoid’s response to environmental changes. This question is addressed here using single DNA manipulation approach. Our results reveal that the two proteins are differentially selected for DNA binding, which can be tuned by changing environmental factors over physiological ranges including KCl (50–300 mM), MgCl_2_ (0–10 mM), pH (6.5–8.5) and temperature (23–37 °C). Increasing pH and MgCl_2_ concentrations switch from Dps-binding to IHF-binding. Stable Dps-DNA and IHF-DNA complexes are insensitive to temperature changes for the range tested. The environment dependent selection between IHF and Dps results in different physical organizations of DNA. Overall, our findings provide important insights into *E. coli* nucleoid architecture.

Bacteria have a genomic DNA with a contour length that can be in the order of millimetre range (e.g. *E. coli*, Salmonella, etc). This is approximately a thousand fold longer than the dimension of a single cell (typically <1 μm)^1^. Packaging of genomic DNA is mediated by a set of DNA-binding proteins abundantly present in the bacterial nucleoid; these proteins are known as nucleoid-associated proteins (NAPs)^2^. Nucleoid architectures determined by NAPs have a significant impact on gene transcription and DNA replication^3^,^4^. Overall, NAPs are critical DNA binding proteins that serve multifunctional roles in bacteria.

Bacteria are frequently exposed to severe environmental changes in pH, temperature, osmolarity and oxidative stress, as well as nutritional deprivation. Under these stressful conditions, the nucleoid in bacteria is organized differently by certain NAPs which also protect the bacterial genome and regulate transcription in order to survive through these growth-limiting and potentially lethal conditions. For example, a recent study has shown that Dan (previously known as TtdR or YgiP) protein is up-regulated 10-fold under anaerobic conditions; it forms a rigid periodic nucleoprotein filament in the absence of magnesium that strongly restricts DNA accessibility and mediates DNA condensation in the presence of physiological levels of magnesium^5^,^6^. Similarly, during nutritional deprivation, DNA-binding proteins from starved cells (Dps), another NAP, is up-regulated to organize the nucleoid and protect it from diverse damages^7^.

The growth of bacteria can be divided into several different phases. When there are plenty of nutrients, bacteria undergo exponential growth. When there are insufficient essential nutrients and/or under other growth-limiting factors, bacteria enter the stationary growth phase where growth rate and death rate are equal. The abundance of different bacterial NAPs species is differentially controlled in different growth phases, resulting in different nucleoid architectures^2^, and affecting gene expression on a global scale. Many studies have focused on understanding the nucleoid architecture in the exponential growth phase, where nucleoid is organized by several major NAPs including factor for inversion stimulation (Fis), heat-unstable nucleoid protein (HU), and histone-like nucleoid structuring protein (H-NS) like proteins^2^.

The DNA binding properties of these proteins have been extensively studied: Fis organizes DNA through DNA bending and DNA condensation by juxtaposition of remotes DNA sites together^8^,^9^; HU is able to bend DNA at low HU concentration and stiffen DNA at high HU concentration, where the switching in binding modes also depends on surrounding osmolarity^10^,^11^. H-NS, a unique global gene-silencing NAP, polymerizes along DNA into an extended rigid nucleoprotein filament favoured at <150 mM potassium and <2 mM magnesium ions concentration^12^,^13^,^14^. At >5 mM magnesium ion concentration, it promotes DNA bridging which could either mediated by interaction between a nucleoprotein filament with another DNA segment^15^ or/and by diffusive H-NS dimers each providing two DNA binding sites^16^. The *E. coli* nucleoid in the exponential growth phase is largely determined by competitive binding of these proteins onto different regions of DNA^17^,^18^.

A major reorganization of the *E. coli* nucleoid occurs upon entering into the stationary phase during nutritional deprivation. It is marked by a transformation from a dispersed morphology into a highly condensed, ordered assembly of ring-shaped structure in the cell centre^19^. During the early stationary growth phase, Dps is the most abundant NAP, followed by Integration host factors (IHF), with a Dps:IHF ratio of around 10:1^20^,^21^, while the expression levels of other NAPs are substantially reduced^22^. At present, how Dps and IHF proteins determine the *E. coli* nucleoid structure in the early stationary growth phase remains unclear. To gain insights into this question, the individual DNA binding properties of Dps and IHF, as well as their competition for DNA binding in different environments must be understood. This is in contrast to studies where the DNA binding modes of Dps and IHF under certain environments were studied individually.

Dps protein had been shown to be able to bind onto DNA without apparent sequence specificity^20^. It not only plays a global regulatory role in controlling gene expression but also a protective role during stationary phase. Dps exists as a compact shell-like dodecameric structure of 12 identical subunits with a hollow iron storage compartment and lysine rich N-terminus of its monomers protruding out^23^,^24^. It binds DNA in a low salt and low pH environment. As pH increases beyond the physiological range, protruding lysine residues are deprotonated, resulting in loss of DNA binding^25^. At higher salt concentrations, DNA binding by Dps is also weakened, due to electrostatic screening between protruding lysine residues and DNA^25^,^26^. Remarkably, the unique dodecameric structure of Dps becomes unstable when temperature is increased to above 65 °C; but pre-formed Dps-DNA complexes are stable against acute heat shock to 100 °C and prolonged heating at 65 °C^20^. Previous atomic force microscopy (AFM) imaging experiments have shown that binding of Dps can organize DNA into highly condensed nucleoprotein complexes^25^. In this study, these previous results obtained by biochemical assays or AFM imaging are re-examined by single-DNA stretching to obtain new insights into the stability of the folded DNA-Dps complex.

IHF was originally discovered as an essential co-factor for site-specific recombination of phage lambda in *E. coli*, and also known as a transcription factor^27^. However, the intracellular concentration of IHF is at least two orders of magnitude higher than its site-specific *k*_D_ of around 1 nM^28^,^29^, suggesting that IHF may associate with DNA in a non-specific manner thus contributing to bacterial chromatin organization^30^,^31^. Indeed, we recently reported that IHF non-specifically binds to DNA in multiple distinct modes in response to solution conditions, resulting in various physical organizations of DNA such as local bending^31^,^32^ and global DNA condensation^32^. In contrast to Dps, IHF reacts oppositely to the presence of MgCl_2_. IHF can bend and introduce a kink to DNA at low KCl concentration in the absence of magnesium; in the presence of MgCl_2_ in millimolar range, IHF mediates DNA cross-linking and aggregate DNA into higher-ordered nucleoprotein complexes^32^.

It is believed that NAPs do not simply play their roles of biological function separately but rather work in tandem with intricate interplay. As Dps and IHF co-exist abundantly during stationary phase and respond differently to changes in environmental factors, it is likely that their relative association to nucleoid is tuned by changes in environmental conditions. It has not been investigated previously on DNA packaging in a mixture containing Dps and IHF at their physiological stoichiometric ratio and its dependence on environmental factors. In this work, this question is systematically examined at a single-DNA level using single-DNA stretching method.

## Results

### Effects of Dps on DNA probed by single-DNA stretching experiments

**Figure 1:**
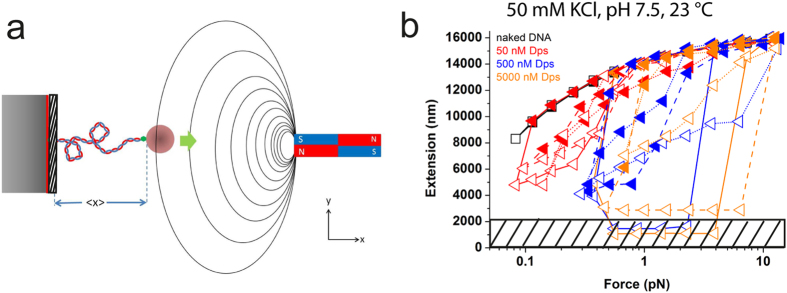
Transverse magnetic tweezers setup and Dps mediated DNA condensation. (**a**) Figure shows an illustration of single-DNA stretching experiment using magnetic tweezers. One biotinylated DNA end is attached to a streptavidin functionalised coverslip and the other biotinylated DNA end attached to a streptavidin coated paramagnetic bead. In the shaded area of 2 micrometers from the coverslip edge, bead image cannot be obtained. Force is exerted by using a permanent magnet and force is adjusted by moving the position of magnet. At different forces, the corresponding extension of DNA is recorded. (**b**) Force-extension curves obtained on a 48,502 bp λ-DNA during force-decrease (solid symbols) and subsequent force-increase scan (open symbols) before and after incubated with 50 nM Dps, 500 nM Dps and 5000 nM Dps in 50 mM KCl, pH 7.5 at 23 °C. Black data shows force extension curve of naked DNA as control where force-decreased and force increased curves overlapped. The black solid curve is a fitting curve by the Worm-like chain DNA polymer model with a persistence length of 50 nm using the Marko-Siggia formula^35^,^36^. Force extension curves after protein incubation are plotted with coloured symbols, coloured lines are connecting lines between each data points for better presentation. Data obtained from three independent experiments at each concentration are shown in the same colour indicated by solid, dashed, and dotted connecting lines. The non-overlapping force-decreased and force increased curves shows that force-extension curves of the DNA interacting with Dps do not reach equilibrium at our force-scanning experimental time scale.

DNA-binding proteins can affect the mechanics of DNA, resulting in changes to the force-extension curves of DNA. Therefore, proteins binding to DNA can be detected at a single-DNA level by probing their effects on the force responses of DNA as previously described and illustrated in Fig. 1^13^,^14^,^31^,^33^. Compared to traditional biochemical detection methods such as electrophoretic mobility shift assay (EMSA), single-DNA stretching provides single-molecule sensitivity and can be done on much longer DNA molecules in varying solution conditions. It also gives information of DNA distortions, such as bending, folding or stiffening, introduced by protein binding^33^.

In this work, we examined the mechanical effects of Dps and IHF binding to a single 48,502 bp λ-DNA using a transverse magnetic tweezers setup revised from that described in our previous work^34^ (Fig. 1a). The two DNA ends are tethered between a coverslip edge and a paramagnetic bead through biotin-streptavidin interaction. Force experienced by the paramagnetic bead is exerted by using a pair of permanent magnets and adjusted by changing the distance of magnets from the bead. The extension of the DNA stretched at various forces can be plotted in a force-extension curve for analysis. At each experiment, prior to introducing proteins, the naked DNA force-extension curve was recorded (Fig. 1b, black symbols). At each force, the DNA was held for 30 seconds and the average extension during the time window is plotted. The force scanning was first started with a force-decrease scan (solid symbols) followed by a force-increase scan (open symbols). In our graph shown, curves obtained during force-decrease and force-increase overlap, indicating the force-extension curve measurements for naked DNA are in equilibrium over the time scale of force-scanning. The naked DNA data in Fig. 1b (black) were confirmed to be from a single DNA tether by fitting with the worm-like chain (WLC) DNA polymer model with a bending persistence length of ~50 nm using the Marko-Siggia formula^35^,^36^ (Fig. 1b, black fitted line). It is noted that due to the shadow of the coverslip edge, we can only measure DNA extension to a minimum value of around 2,000 nm; any length shorter than the minimum are not included in our data analysis.

Figure 1b shows the force-decrease and force-increase curves obtained at 50 mM KCl, pH 7.5 and 23 °C with increasing concentration of Dps over a wide range of 50–5,000 nM. In the presence of Dps, the force-decrease and force-increase curves are not overlapping (hereafter referred to as “hysteresis”), with the force-increase curve below the preceding force-decrease curve. These results suggest that Dps mediates DNA folding at low forces and that the interaction did not reach equilibrium over our force-scanning time scale. In all our subsequent experiments, a fixed concentration of 500 nM Dps was used to investigate the effects of various environmental factors on the DNA binding by Dps. Due to the non-equilibrium nature of the Dps-mediated DNA folding; the level of hysteresis depends on the force-scanning time scale. In this work, we used a fixed scanning speed to probe DNA binding by Dps and IHF as well as their competition in different solution conditions.

We first probed Dps binding to DNA in different potassium and magnesium concentrations. The DNA binding properties of Dps at different salt concentrations has been extensively studied previously by biochemical assays. Here they are re-examined by single-DNA stretching based on their mechanical effects, which forms the basis for subsequent studies of competitive binding with IHF in response to environmental changes. Dps binds to DNA based on electrostatic interaction and this is regulated by ionic strength. Besides, previous studies suggested that the Dps-DNA complex formation is mediated through multiple ion bridges that are maintained by doubly charged cations; therefore Dps binding should also be sensitive to MgCl_2_ concentration^22^,^25^.

In *E. coli*, potassium ion is a primary intracellular ionic osmolyte which can be changed over a wide range in response to change in osmolarity of the external growth medium^37^,^38^. Its concentration is estimated to be in the range of 50–300 mM^39^, which is much more abundant than sodium ion which concentration is tightly regulated at <10 mM concentration^40^,^41^. In order to understand how DNA binding by Dps is affected by potassium ions, we recorded the force-extension curves of DNA in 50 mM and 150 mM KCl at pH 7.5 at 23 °C.

**Figure 2:**
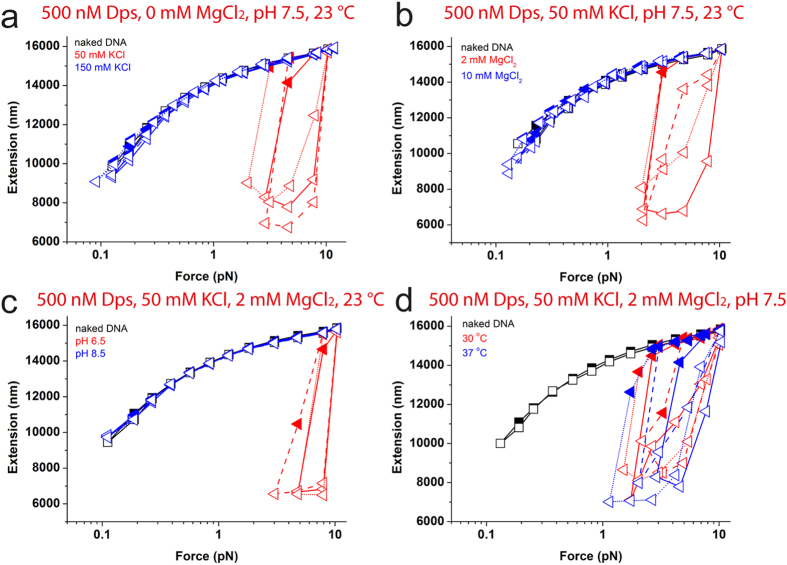
Environment dependence of Dps mediated DNA condensation. (**a-d**) Black symbols show force-extension curves of naked DNA as comparison, force-decreased scan shown in solid symbols and subsequent force-increased scan shown in open symbols. Force-extension curves in the presence of 500 nM Dps in increasing concentration of KCl (**a**), in 50 mM KCl with increasing concentration of MgCl_2_ (**b**), in 50 mM KCl and 2 mM MgCl_2_ at different pH (**c**) and at different temperature at pH 7.5 (**d**). Data from three independent experiments for each condition are included in each figure panel in the same colours but different connecting lines.

At 50 mM KCl, large hysteresis due to Dps mediated DNA folding between force-decrease and force-increase curves was observed in >5 independent experiments. Figure 2a shows three independent data sets in red colour for clarity. However, at 150 mM KCl (Fig. 2a, blue) or higher concentration (data not shown), the force-extension curves overlap with the naked DNA curve, indicating that Dps losses the capability of folding DNA, which can be explained by electrostatic screening between the protruding lysine residues and DNA at higher monovalent salt concentrations.

Magnesium ion is essential in bacteria for many enzymatic reactions and the *in vivo* concentration is in the low mM range^42^,^43^. The effects of magnesium ion on Dps binding were examined at 0 mM, 2 mM and 10 mM MgCl_2_ concentrations in 50 mM KCl, pH 7.5 at 23 °C. Force-extension curves of DNA incubated with 500 nM Dps in the absence of magnesium ions had been previously showed in Fig. 2a (red), indicating a DNA folding. In 2 mM MgCl_2_ (Fig. 2b, red), DNA folding was still observed. Further increase in MgCl_2_ concentration weakens Dps-mediated DNA folding. At 10 mM MgCl_2_, Dps-mediated DNA folding was no longer observed and the DNA behaved almost like a naked DNA with the absence of hysteresis between the force-decrease and force-increase curves (Fig. 2b, blue). Therefore we conclude that Dps-DNA binding is also sensitively regulated by MgCl_2_, which is consistent with previous biochemical and AFM studies that reported loss of DNA aggregation in the presence of Dps in high magnesium concentrations^25^,^26^.

Besides potassium and magnesium ion concentrations, there are other important environmental factors which also affect cell growth and function, such as pH and temperature. Data in Fig. 2c shows force extension curves in 500 nM Dps in 50 mM KCl and 2 mM MgCl_2_ at different pH values. Dps-mediated DNA folding at pH 6.5 (Fig. 2c, red) is more significant than that at pH 7.5 shown previously (Fig. 2b, red), indicated by significantly larger forces where DNA folding started. Further increasing the pH to 8.5 results in Dps failing to fold DNA (Fig. 2c, blue). These results are consistent with previous AFM studies that showed Dps-mediated DNA condensation is eliminated by increasing pH^25^.

The effects of temperature were investigated similarly in 500 nM Dps, 50 mM KCl and 2 mM MgCl_2_ at pH 7.5. We found that temperature has little influence on DNA folding by Dps (Fig. 2d).

### Effects of changes in environmental factors on IHF-DNA complex

Before we address the competitive DNA binding between Dps and IHF in various environments, we determined the force responses of DNA to IHF binding. Non-specific binding of IHF to DNA and its dependence on MgCl_2_ concentrations were investigated recently, which revealed that IHF binds and bends DNA in the absence of MgCl_2_^32^. In the presence of MgCl_2_ of 2 mM or greater, IHF binding to DNA could bring remote DNA sites together inducing DNA condensation^32^. The effects of temperature and pH have not been studied. Here the effects of temperature and pH as well as MgCl_2_ were examined using the same experimental procedure as in the studies of Dps-DNA interaction. The *in vivo* stoichiometric ratio of Dps:IHF is about 10:1^22^; therefore we chose a fixed concentration of 50 nM IHF in the subsequent experiments which is 10 times smaller than the Dps concentration used previously.

**Figure 3:**
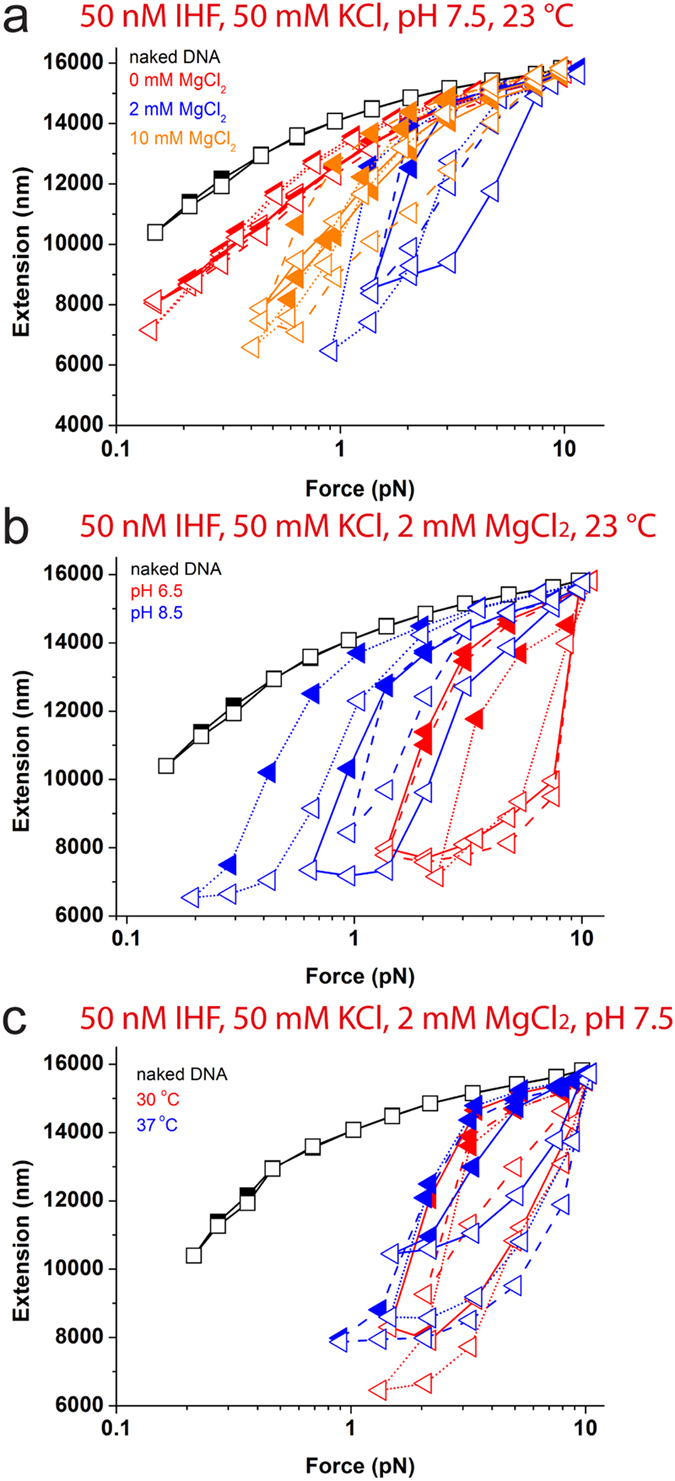
Effects of MgCl_2_ concentration, pH and temperature on IHF-DNA complex. (**a-c**) Black symbols show force-extension curves of naked DNA as comparison. Coloured symbols show force-extension curves in 50 nM of IHF in 50 mM KCl at increasing magnesium concentrations (**a**), in 50 mM KCl and 2 mM MgCl_2_ at increasing pH values (**b**) and increasing temperatures at pH 7.5 (**c**). Three representative independent experiments for each condition are included in each figure panel in the same colours but different connecting lines.

Figure 3a shows representative force-extension curves of IHF-DNA complex in buffer solution containing 50 nM IHF, 50 mM KCl and pH 7.5 at 23 °C with increasing magnesium concentration. In the absence of magnesium, the force-decrease and force-increase curves overlap, indicating that IHF binding to DNA has reached equilibrium at our force-scanning speed (Fig. 3a; red). The DNA extension is shorter than that of the naked DNA at low force, which is expected from DNA bending by IHF, consistent with previous study^31^. At increased MgCl_2_ concentrations of 2 mM (Fig. 3a, blue), besides being shorter than the naked DNA, the force-decrease curves (solid symbols) are significantly longer than the subsequent force-increase curves (open symbols), which is consistent with IHF mediated DNA folding in the presence of MgCl_2_ as shown in our previous studies^32^. IHF mediated DNA folding was also observed at 10 mM MgCl_2_ (Fig. 3a, orange); however, hysteresis between force-decrease and force-increase curves are less significant compare to that obtained at 2 mM MgCl_2_. We reason that it is likely due to the increased electrostatic screening effect at the higher MgCl_2_ concentration.

Similar experiments were done in 50 mM KCl and 2 mM MgCl_2_ with varying pH values from 6.5 to 8.5. At all pH conditions, IHF mediated DNA folding occurred as indicated by hysteresis between the force-decrease and force-increase curves (Fig. 3b, red; Fig. 3a, blue; Fig. 3b, blue).

The effect of temperature on DNA binding by IHF was investigated similarly in 50 mM KCl and 2 mM MgCl_2_. At 23 °C (Fig. 3a, blue), 30 °C (Fig. 3c, red) and 37 °C (Fig. 3c, blue), similar degrees of hysteresis induced by IHF mediated DNA folding were observed, indicating that the IHF-DNA complex is insensitive to temperature changes.

Overall, our data revealed that interactions of Dps and IHF with DNA molecules differentially depend on environmental factors. Dps is able to bind DNA at low KCl and MgCl_2_ concentration, as well as low pH, resulting in DNA folding. The DNA binding property of Dps is not affected by temperature change over physiological range. On the other hand, IHF switches its DNA-binding mode from bending to folding upon the addition of MgCl_2_. However, IHF-DNA interactions are not affected by pH and temperature changes.

### Environment-dependent competitive binding of Dps and IHF to DNA

Differential responses of Dps and IHF DNA binding properties to environmental changes form the basis for analyzing their competitive binding to DNA. We next investigated the dynamic interaction of a mixture of IHF and Dps with DNA to understand how competitive binding of these two proteins is affected by various solution conditions. The *in vivo* stoichiometric ratio of Dps:IHF is about 10:1^22^. To study competitive binding, we incubated DNA with a mixture containing 50 nM of IHF and 500 nM of Dps in various solution conditions.

**Figure 4:**
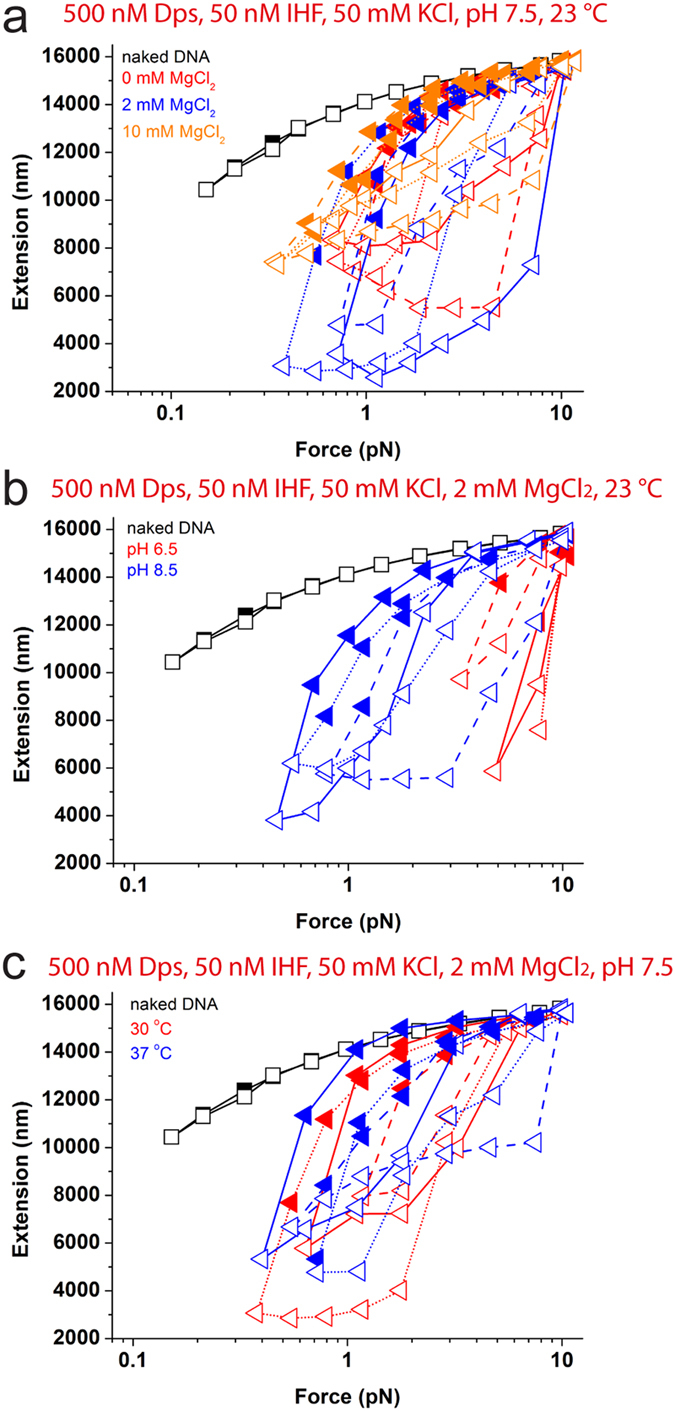
Selection of Dps and IHF by DNA as an effect of changes in surrounding environment. (**a-c**) Black symbols show force-extension curves of naked DNA as comparison. Coloured symbols show force-extension curves obtained with a mixture of 50 nM IHF and 500 nM Dps in 50 mM KCl at increasing MgCl_2_ concentration (**a**), in 50 mM KCl and 2 mM MgCl_2_ at increasing pH value (**b**) and increasing temperature at pH 7.5 (**c**). Multiple repeating experiments for each condition are included in each figure panel in the same colours but different connecting lines.

Figure 4a shows the force-extension curves after incubating the DNA with the protein mixture at 50 mM KCl, pH 7.5 at 23 oC and varying magnesium ion concentration. In the absence of magnesium, folding of DNA with hysteresis was observed (Fig. 4a, red) and this folding must be due to binding of Dps onto DNA, as in such condition IHF alone only bends DNA. However, IHF binding may also occur concurrently, but its effect on DNA cannot be detected in the folded DNA. At 2 mM MgCl_2_, where both IHF and Dps were able to bind and fold DNA, similar level of DNA folding is observed (Fig. 4a, blue). Increasing to 10 mM MgCl_2_, DNA folding was still observed but at a lesser extent indicated by reduced hysteresis (Fig. 4a, orange). Given that at 10 mM MgCl_2_ Dps does not bind DNA (Fig. 2b, blue), this DNA folding should be due to IHF binding. By comparing to the IHF-DNA binding previously done at this condition (Fig. 3a, orange), the level of hysteresis in Fig. 4a is slightly more pronounced compared to that in Fig. 3a, but the difference is not significant. Based on these results, we conclude that increasing MgCl_2_ concentration can switch DNA binding from both Dps and IHF to IHF alone, both resulting in DNA folding but at slightly different levels and by different mechanisms.

Similar experiments were conducted to investigate the effects of increasing pH in 50 mM KCl and 2 mM MgCl_2_ at 23 oC. At pH 6.5 (Fig. 4b, red), DNA folding occurred at forces slightly below 10 pN, which is significantly larger than that observed at pH 7.5 (Fig. 4a blue), and 8.5 (Fig. 4b, blue). Note that DNA folding at pH 6.5 and 7.5 could be due to DNA binding of both IHF and Dps, while DNA folding at pH 8.5 could only be caused by IHF binding alone. Overall, these results indicate DNA compaction by both IHF and Dps and a switch to selective binding of IHF alone during increased magnesium ion concentration and decreased acidity (increased pH). Hence, DNA is able to retain its condensation state during pH change over a physiological range.

As shown previously in figures 2d and 3c, both Dps and IHF mediated DNA folding remained stable in the temperature range of 23–37 °C. This predicts that in the mixture of Dps and IHF, DNA condensation state will always be attained, by binding of Dps, IHF or both, depending on surrounding salt concentration and pH condition. Consistent with this prediction, we found that in 500 nM Dps and 50 nM IHF at 50 mM KCl, 2 mM MgCl_2_ and pH 7.5, the force-extension curves demonstrate large scale DNA folding with strong hysteresis when temperature was increased from 23 °C to 37 °C (Fig. 4a, blue; Fig. 4c, red and blue).

**Figure 5:**
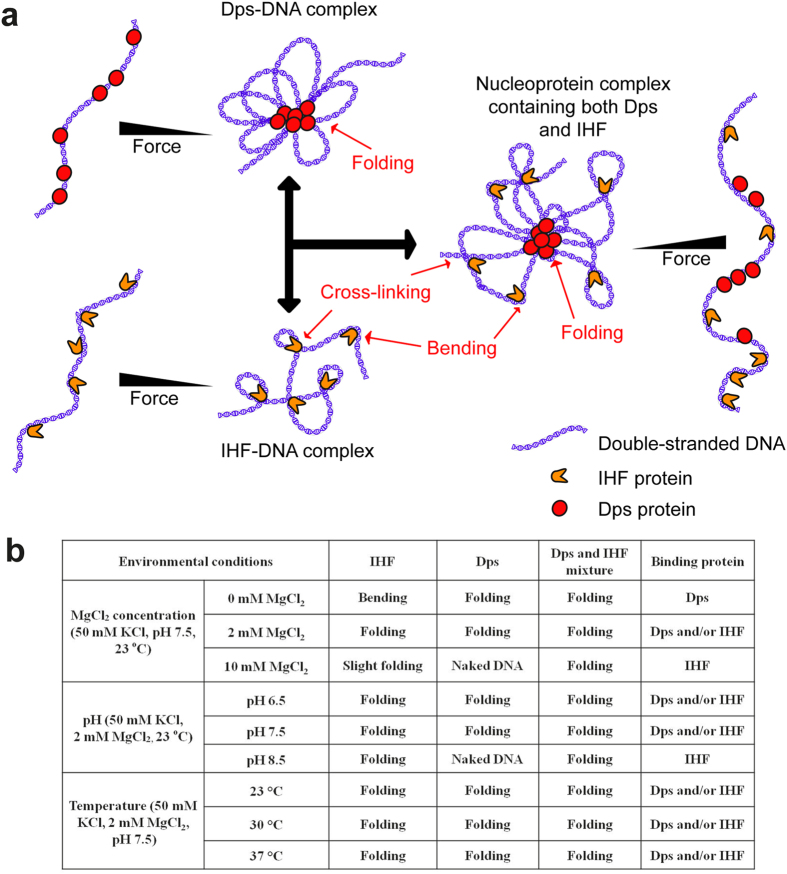
(**a**) Schematic showing different DNA binding modes of Dps or/and IHF proteins. At high force, Dps binds onto DNA but cannot wrap/condense DNA. Upon decreasing the force, these proteins are able to interact with each other and hence result in cooperative condensation of the DNA as reported in previous studies^25^. Meanwhile for IHF protein, at high force it binds but cannot bend the DNA. Upon decreasing the force, IHF bends DNA at the binding sites, and are able to juxtapose remote DNA sites together in the presence of magnesium^32^. Putting these two scenarios together, in physiological solution condition, a nucleoprotein complex containing both Dps and IHF proteins likely form more complex higher order structure mixed with DNA condensation, juxtaposition and DNA bending at low forces, which can be unravelled by increasing force. (**b**) Table showing selective DNA binding of proteins Dps and IHF in different environmental conditions and the resulting DNA conformations.

In summary, our data reveal that selected binding of Dps, IHF, or both to maintain different types of DNA condensation depends on the environment. A schematic of the protein-DNA binding are shown in Fig. 5a, according to the known structural and biochemical information about the proteins and their complexes with DNA. At high force, Dps and IHF both can binds onto DNA individually but not able to condense or bend DNA. When force is decreased, Dps are able to interact with each other and cooperatively condense the DNA as reported in previous studies^25^. As for IHF, it is able to bend DNA at the binding sites in the absence of magnesium and to juxtapose remote DNA sites together in the presence of magnesium. Therefore, putting these two scenarios together, a nucleoprotein complex containing both Dps and IHF proteins likely form a more higher order structure mixed with DNA condensation, juxtaposition and DNA bending at low forces, which can be unravelled by increasing force. A summary of the binding of Dps and IHF to DNA individually, as well as the preferential selection of protein for DNA-binding in different environmental conditions and their resulting DNA conformations are summarized in a table form in Fig. 5b.

## Discussion

Results from this work have revealed several interesting phenomena regarding how DNA binding properties of the two most abundant nucleoid associated proteins in the early stationary phase of *E. coli*, Dps and IHF, respond to major environmental changes. We find that these two proteins respond to environmental changes in markedly different manners. Dps binding is impaired by increasing pH or KCl and MgCl_2_ concentrations, while change in temperature over a physiological range has little influence. In contrast, increasing MgCl_2_ or acidity does not switch off IHF binding; rather, it changes the IHF binding mode from DNA bending to global DNA condensation through juxtaposition of remote DNA sites in the presence of magnesium ions. Here we note that IHF-mediated DNA condensation at high acidity has not been reported before. Also, IHF binding on DNA is insensitive to temperature in the range tested. Regarding the dependence on KCl concentration, we have shown in our previous work^32^ that IHF binding is sensitive to increasing KCl concentration, which is similar to the KCl dependence of DNA binding by Dps.

The differential environmental responses between Dps and IHF suggest that, in a solution with both proteins, they can be preferentially selected for DNA binding at different environments, thus resulting in environmentally dependent nucleoid organization. A bacterial has the same temperature as the surrounding environment^44^, and its intracellular potassium ion concentration increases as external osmolarity increases^37^,^38^. The acidity of the cytoplasm of bacteria also changes in response to extracellular acid stress^45^. The *E. coli* cytoplasm pH rapidly falls when it is subject to extracellular acid stress. Some cells recovers to pH 7.0 or greater, but some do not^46^. Although the regulation of free magnesium ions concentration is not clear, it is believed to vary within the range of a few mM^42^,^43^. Therefore, our studies of the selective binding of IHF and Dps in response to changes of these factors can provide insights into the interplay between IHF and Dps in DNA binding and chromosome organization.

As summarized in the table in Fig. 5b, over the ranges of environmental factors tested in the study, DNA remains in a compact state by binding of IHF, or Dps, or both. This suggests that *E. coli* may retain its capability of changing its nucleoid architecture in rapid response to environmental changes while keeping the nucleoid in a compact state. It has been known that Dps is responsible for nucleoid packaging during the stationary phase. The results from this work suggest that both Dps and IHF are likely involved in nucleoid packaging during the early stationary phase. As mentioned above, abundant NAPs during exponential growth phase, such as Fis, Hu, H-NS, and IHF, have distinct DNA binding properties and different environmental dependency^8^,^9^,^10^,^11^,^13^,^14^,^30^,^31^,^32^,^47^. Therefore, we anticipate that similar preferential selection of nucleoid associated proteins with environmental dependency also controls bacterial chromosome packaging at the exponential growth phase.

According to previous studies, the IHF dependent DNA condensation is based on a juxtaposition of remote DNA sites together in the presence of magnesium^32^, while Dps mediated DNA condensation has been suggested to be based on formation of stacked alternating layers that leads to formation of large Dps-DNA co-crystals^48^. Our results imply that both IHF and Dps may together bind to the nucleoid in most of the physiological relevant conditions, which may result in complex chromosome organization because of their different DNA architectural properties in an environment dependent manner.

It has been known that NAPs are global transcription factors, regulating gene transcription in different ways. Dps has been known to protect the condensed DNA from being damaged and also causes global gene silencing^20^. IHF also affects gene transcription globally. Previous studies have reported that IHF affects gene expression levels, with 42 upregulated and 39 downregulated^49^. The activation of gene transcription of IHF has been suggested to be related to its DNA bending property^50^. As Dps is a global silencer while IHF selectively regulates the transcription of a set of genes, this creates a scenario that Dps and IHF may act together to control the balance of nucleoid gene transcription during the early stationary phase of *E. coli*, which is influenced by changes of environmental factors.

From the result of this work, we hypotheses that in a nutrition-deprived condition, a switch from IHF-binding to Dps-binding could result in global gene silencing; this can reduce the level of metabolism in the cell which in turn save resources. It had been known that when a cell from stationary growth phase is provided with fresh medium with sufficient nutrient, they could move on to exponential phase and is able to continue its metabolic activities^51^. Therefore, upon release of environmental stress, the cell likely has the capability of resetting its nucleoid state. In such a process, IHF may play a crucial role by binding onto DNA and activating a different global gene transcription pattern. Sequence selective binding, which is important to provide further insights to the transcription control by the collective actions of IHF and Dps, is not provided in this work that warrants future studies.

Taking together, the results from this work suggest that *E. coli* is able to organize the nucleoid differentially in response to environmental changes by selecting a subset of available NAPs for DNA binding, which may be important not only for chromosome packaging but also for transcriptional control.

## Methods

### Over-expression and purification of Dps and IHF

The *E. coli* Dps gene sequence is derived from NCBI GenBank (Accession number: CAA49169) and synthesized (1st Base, Singapore). The synthesized gene was cloned into pET vector using NdeI and XhoI (New England Biolabs) for expression and purification. The pET expression vector was chosen such that the DPS protein was expressed with a cleavable N-terminal 6X-HIS-tag. The expression and purification protocols are the same as previously described 20. The purified HIS-tagged DPS protein was then cleaved with thrombin to yield the native DPS protein and dialyzed against 10 mM tris, pH 7.4, 500 mM KCl before storing in final 30% glycerol condition at 20 °C. The gene synthesis derived Dps protein was finally verified with mass spectroscopy.

Purified *E. coli* wild-type IHF was a kind gift of D. Esposito to P.D., which was expressed and purified according to the original protocol from Howard Nash^52^.

### Magnetic tweezers experiments

The magnetic tweezers setup was similar to that used in our previous studies^6^,^14^,^34^. A single λ-DNA of 48502 base pairs was biotin-labelled at both ends. One end of the biotinylated DNA was tethered to a streptavidin functionalized surface, and the other end to a streptavidin-coated magnetic bead (Dynabeads® M-270 Streptavidin, Invitrogen). Figure 1a shows the schematic diagram of the magnetic tweezers setup. Real time extension measurement of the DNA molecule was recorded using a camera-based centroid tracking software written in LabVIEW program (National Instruments, U.S.A). In the magnetic tweezers experiment, only one physical variable of the nucleoid architecture is measured, that is the end-to-end distance of the DNA. This reading is plotted against force in the force-extension curve, and the protein binding is detected by the resulting changes in the force-extension curve. Note that in all our force-extension curves in the result section, the coloured lines do not represent a fitting; they are merely connecting lines for better presentation.

## Additional Information

**How to cite this article**: Lee, S. Y. *et al.* Regulation of Bacterial DNA Packaging in Early Stationary Phase by Competitive DNA Binding of Dps and IHF. *Sci. Rep.* **5**, 18146; doi: 10.1038/srep18146 (2015).
